# Rapid Antigen Test LumiraDx^TM^ vs. Real Time Polymerase Chain Reaction for the Diagnosis of SARS-CoV-2 Infection: A Retrospective Cohort Study

**DOI:** 10.3390/ijerph19073826

**Published:** 2022-03-23

**Authors:** Anna Maria Cattelan, Lolita Sasset, Federico Zabeo, Anna Ferrari, Lucia Rossi, Maria Mazzitelli, Silvia Cocchio, Vincenzo Baldo

**Affiliations:** 1 Infectious and Tropical Diseases Unit, Azienda Ospedale Università di Padova, 35128 Padova, Italy; lolita.sasset@aopd.veneto.it (L.S.); anna.ferrari@aopd.veneto.it (A.F.); maria.mazzitelli@aopd.veneto.it (M.M.); 2 Department of Cardiac Thoracic and Vascular Sciences and Public Health, University of Padova, 35127 Padova, Italy; federico.zabeo@unipd.it (F.Z.); silvia.cocchio@unipd.it (S.C.); vincenzo.baldo@unipd.it (V.B.); 3 Microbiology Department, Azienda Ospedale Università di Padova, 35128 Padova, Italy; lucia.rossi@aopd.veneto.it

**Keywords:** SARS-CoV-2, COVID-19, diagnosis, rapid antigen testing, RT-PCR, agreement, sensitivity, specificity, positive predictive value, negative predictive value, accuracy

## Abstract

Background: Real time reverse transcription polymerase chain reaction (real time RT-PCR) testing is the gold standard for the diagnosis of SARS-CoV-2 infections. However, to expand the testing capacity, new SARS-CoV-2 rapid antigen tests (Ag-RDTs) have been implemented. Ag-RDTs are more rapid, but less reliable in terms of sensitivity, and real-life data on their performance in comparison with the real time RT-PCR test are lacking. Methods: We aimed at assessing the diagnostic performance of the third-generation antigenic swab LumiraDx™ compared with real time RT-PCR in a retrospective cohort study at the Infectious Diseases Unit of Padua. All of the patients who were consecutively tested for SARS-CoV-2 in our centre (by both real time RT-PCR and Ag-RTD LumiraDx^TM^) from 19 January to 30 May 2021, were included. Cycle-threshold (Ct) values of positive real time RT-PCR were recorded as well as the number of days from symptoms’ onset to testing. Results: Among the 282 patients included, 80.9% (N = 228) tested positive to real time RT-PCR, and among these, 174 tested positive also to LumiraDx™. Compared with real time RT-PCR, which is considered as the gold standard for the assessment of the presence/absence of SARS-CoV-2 infection, LumiraDx™ showed an overall sensitivity of 76.3% and specificity of 94.4%. Sensitivity increased to 91% when testing was performed <10 days from symptoms’ onset, and to 95% when considering Ct < 25. Multivariable binomial logistic regression showed that false negative LumiraDx™ results were significantly associated with high Ct values, and with further testing from symptoms’ onset. Conclusions: The results of our study suggested that the LumiraDx™ SARS-CoV-2 antigen assay may be appropriate for the detection of SARS-CoV-2 infection, especially in its early phase when the test largely meets the performance requirements of the European Centre for Disease Prevention and Control (ECDC).

## 1. Introduction

The outbreak of Coronavirus disease 2019 (COVID-19) was first recorded in Wuhan (China) in late 2019 and rapidly spread worldwide. As of 30 March 2020, it has been officially declared as a global pandemic [1]. Despite unprecedented public health interventions, the transmission of severe acute respiratory syndrome coronavirus 2 (SARS-CoV-2) is still ongoing. The early detection of new infections impacts patient management and the transmission of SARS-CoV-2 in the community.

The current gold standard for the microbiological diagnosis of SARS-CoV-2 is the detection of its genetic targets in respiratory samples using the molecular real time reverse transcription polymerase chain reaction (real time RT-PCR) test [2]. Moreover, this test is used for the epidemiological surveillance of infection, aiming at containing the further spread of the virus. As the contagion can occur both through individuals before symptoms’ onset and even through those who never develop symptoms, extensive community testing is considered as a cornerstone of infection control strategies [3].

Despite the high sensitivity and specificity, the real time RT-PCR test suffers from a series of limitations, such as the long time it takes for performance and processing, the need for dedicated equipment and highly specialised laboratory technicians, and high costs. For these reasons, SARS-CoV-2 Ag-RDTs are considered as a complement to PCR-based testing [4]. They are commonly based on the lateral flow principle and work with respiratory specimens, such as nasopharyngeal swabs. In contrast to PCR-based testing, they do not require special training and are able to provide results in a few minutes. As easy-to-use emerging diagnostic tools, Ag-RDTs have the potential to play a significant role in guiding patient management, public health interventions, and disease surveillance. Numerous commercial assays are now available, but there are still limited data on their clinical performance [5,6,7]. In accordance with previous studies, the sensitivity of SARS-CoV-2 Ag-RDTs ranges between 22.9 and 93.9% when compared with real time RT-PCR [8,9,10,11], depending on the stage of infection. A recent meta-analysis demonstrated that the sensitivity of Ag-RDTs was 50.7% (35.6–65.8%) among subjects with cycle-threshold (Ct) values ≥ 25, whereas it increased to 95.8% (92.3–97.8%) when restricted to samples with high viral loads (i.e., Ct values < 25) [12]. At the same time, sensitivity was shown to be 83.8% (76.3–89.2%) when testing was performed in patients within the first week from symptoms’ onset, whereas it fell to 61.5% (52.2–70%) when testing was performed after 1 week from symptoms’ onset [12].

LumiraDx™ (LumiraDx International Ltd., London, UK) has developed a diagnostic device using the microfluidic immunofluorescence technology with an automated read-out to enable the rapid detection of SARS-CoV-2 Ag-RDT from a nasal mid-turbinate swab [13]. In the United States, LumiraDx™ SARS-CoV-2 Ag-RDT was granted under an emergency use authorisation by the Food and Drug Administration (FDA) in August 2020 [14]. Shortly after, in the United Kingdom, a large pharmacy chain offered the LumiraDx™ SARS-CoV-2 test at points of care [15,16]. The test performance, as reported by the manufacturer, demonstrated 97.6% sensitivity and 96.6% specificity mostly among symptomatic individuals within 12 days of symptoms’ onset [13]. These data could largely comply with the WHO guideline requirements for the use of all rapid SARS-CoV-2 tests (a minimum of 80% sensitivity and 97% specificity) [17]. More recently, the ECDC proposed a more conservative threshold of ≥90% for the sensitivity parameter, especially in low-incidence settings [18]. A recent multi-centre manufacturer-independent study showed sensitivity of 82.2% and specificity of 99.3% for the LumiraDx™ microfluidic immunofluorescence assay in a clinical setting [19].

However, in a scenario where there is a dire need for sustained availability of SARS-CoV-2 Ag-RDTs to contain active and future outbreaks, real-life data on the performance of Ag-RDTs are still scarce and not completely conclusive.

Therefore, the purpose of this study was to evaluate the diagnostic accuracy of third-generation antigenic swab (LumiraDx™) compared with the gold standard real time RT-PCR in a retrospective cohort of both symptomatic and asymptomatic adult patients, throughout different clinical stages of the SARS-CoV-2 infection, who came to observation for SARS-CoV-2 testing during the second wave of COVID-19 pandemic in a third-level hospital centre in Northern Italy.

## 2. Materials and Methods

### 2.1. Testing Procedure and Data Collection

We collected 282 respiratory samples from as many individuals who attended the Infectious Diseases Unit of Padua (Italy) for SARS-CoV-2 testing, between January and May 2021. The reasons for testing were the occurrence of symptoms (88.7%) or screening independent of symptoms (i.e., close contact with a SARS-CoV-2 infected subject). Samples from patients already diagnosed with SARS-CoV-2 infection were excluded.

Subjects were included if they received simultaneously two different swabs: The first was for an antigenic nasal test (LumiraDx™), while the second was a nasopharyngeal swab analysed with a real time RT-PCR assay.

The nasopharyngeal swabs for the molecular exams were obtained using flocked swabs in liquid-base collection and transport systems. Samplings were performed in accordance with the United States ECDC guidelines for oropharyngeal/nasopharyngeal testing. The samples were stored at 2–8 °C until testing, which was performed within 72 h from collection. The primers and probes used were tested for the genes encoding the envelope and RNA-dependent RNA polymerase (*RdRp*: RdRp_SARSr-F, RdRp_SARSr-R, RdRP_SARSr-P1, and RdRp_SARSr-P2) of SARS-CoV-2.

Real time RT-PCR assays were performed in a final volume of 25 μL, containing 5 μL of purified nucleic acids, using the One Step Real Time kit (Thermo Fisher Scientific, Waltham, MA, USA) and were carried out on ABI 7900HT Fast Sequence Detection Systems (Thermo Fisher Scientific, Waltham, MA, USA). The sensitivity of *E* and *RdRp* gene assays was 5.0 and 50 genome equivalent copies per reaction at 95% detection probability, respectively. Both assays had no cross-reactivity with the endemic human coronaviruses HCoV-229E, HCoV-NL63, HCoV-OC43, and HCoV-HKU1 with MERS-CoV.

Nasal swabs were performed in accordance with the manufacturer’s instructions: The swab was inserted in both nostrils to a distance of about 2 cm, while gently rotating against the nasal wall several times. Herein, we tested the nostrils and nasal basil. All of the samples were tested in less than 5 h with the LumiraDx™ platform. The detection of SARS-CoV-2 was performed in settings with more than 15 °C. The LumiraDx™ SARS-CoV-2 Ag-RDT is a rapid microfluidic immunofluorescence assay intended for the qualitative detection of nucleocapsid protein antigen to SARS-CoV-2 in nasal swab or nasopharyngeal swab samples. The manufacturer declares no cross reaction with HCoV-229E, HCoV-OC43, HCoV-NL63, and MERS-CoV, as well as with H1N1, H3N2, *Adenovirus*, hMPV, *Enterovirus*, *Respiratory Syncytial virus*, and *Rhinovirus*.

Demographic and clinical characteristics of the tested patients, reasons for SARS-CoV-2 testing, time between SARS-CoV-2 testing and the possible onset of symptoms, and Ct values in positive real time RT-PCR were recorded. All of the tests were performed at the Padova University Hospital.

### 2.2. Statistical Methods

The data were summarised using percentage or mean values with their 95% confidence interval, as appropriate. To compare percentages among the various subgroups, we adopted the chi-square test (possibly with Yates’ correction), whereas to compare mean values, we used the *t*-test.

To assess the diagnostic accuracy of LumiraDx™, we used the following parameters: sensitivity, specificity, positive predictive value (PPV), negative predictive value (NPV), accuracy, and Cohen’s kappa coefficient (K). All of these parameters have been computed (assuming as ground-truth the real time RT-PCR testing results). In particular, the false negative (false positive) LumiraDx™ has been defined as the negative (positive) LumiraDx™, which is disconfirmed by the positive (negative) real time RT-PCR assay. The confidence interval for each of the latter parameters, except for K, was computed through the simple asymptotic formula:

$$p\pm \;z_{1-\frac{\alpha }{2}}\sqrt{\frac{p\left(1-p\right)}{n}}$$

truncated at 0 (on the left) and at 1 (on the right), and then expressed in percentage, where *p* represents the particular estimate of sensitivity, specificity, PPV, NPV or accuracy, for which we are computing the confidence interval, *n* is the numerosity of the subpopulation considered for the computation of *p* (i.e., the number of all molecular positives for sensitivity, the number of all positive tests for PPV, the number of all molecular negatives for specificity, the number of all negative tests for NPV, and finally the numerosity of the whole cohort for accuracy), and $z_{1-\frac{\alpha }{2}}$ is the ($1-\frac{\alpha }{2}$)-percentile for the standard normal distribution, which coincides with 1.96 since we are considering the significance level α = 5%.

On the other hand, an approximate confidence interval for K was computed as K ± $z_{\frac{\alpha }{2}}$ SE(K), where SE(K) denotes the standard error in the estimation of the K coefficient.

Following the first stage of exploratory analysis, we focused on the relation between the false negative results of LumiraDx™ and both the Ct (Ct) values of the tested patient and the number of days elapsed between symptoms’ onset and testing date (Days). To this aim, we restricted our analysis to only symptomatic subjects that tested positive after symptoms’ onset to real time RT-PCR, which account for 78.7% (no. 222) of all our study population. We computed the correlation among continuous variables, such as Ct values and Days, through the ρ coefficient of Spearman’s rank correlation test. We recall that ρ is a value between −1 and 1 and gives a non-parametric measure of the rank correlation between two variables, which assesses how well the relationship between these two latter variables can be described using the monotonic function.

Finally, we performed multivariable binomial logistic regression, considering the discordance between the LumiraDx™ outcome and the consecutive positive real time RT-PCR (false negative) as a dependent variable, and the sex or age group, Days, and Ct values as independent covariates. For Ct values and Days, to make the outcome more readable, we split the study population in a few numbers (three) of macro groups.

Data collection, data cleaning, data visualisation, and statistical analysis were carried out with Excel^®^ 2013 (Microsoft Corporation, Albuquerque, NM, USA) or statistical packages from Python 3.7.0 (Python Software Foundation, Fredericksburg, TX, USA), as appropriate. The multivariate binomial logistic regression was conducted using SPSS 27.0 (IBM, Armonk, NY, USA).

## 3. Results

### 3.1. Study Population

Over the study period, 282 individuals were consecutively tested for SARS-CoV-2 both by real time RT-PCR and the third-generation antigenic swab LumiraDx™ at the Infectious and Tropical Diseases Unit of the teaching Hospital of Padua, Italy. The mean age of the tested population was 61 (±18) years and mainly of male gender (62.8%). Thirty-two tests (11.3%) were performed on asymptomatic subjects, whereas the remaining (88.7%) were performed on symptomatic individuals. The majority (58.4%) of symptomatic individuals complained of minor symptoms, 40.8% were diagnosed with pneumonia, and two suffered from COVID-19-related neurological symptoms.

**Figure 1:**
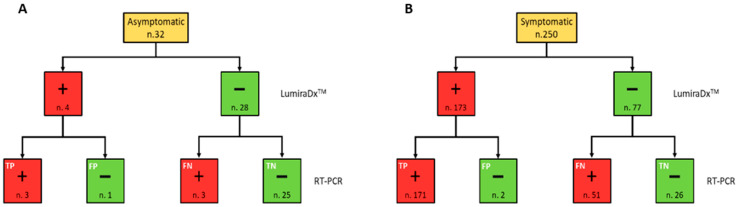
Flow chart summarising the RT-PCR and LumiraDx™ SARS-CoV-2 simultaneously performed test results, in accordance with the symptomatic (**A**) and asymptomatic (**B**) clinical status of the study population. Red and green boxes indicate positive (+) and negative (−) results for each testing, respectively.

The positivity rate did not present significant differences (*p* = 0.22) between males and females. On the other hand, the age group 60–79 years presented a significantly greater positivity rate than both age groups 40–59 and 0–39 years (*p* = 0.005 and *p* < 0.001, respectively). Moreover, the positivity rate was significantly higher both in subjects with symptoms than in asymptomatic (*p* < 0.001) and those diagnosed with pneumonia (*p* = 0.001). Among the 282 subjects who were tested, the outcome of LumiraDx™ turned out to be false negative (FN) in 54 cases and false positive (FP) in only 3 cases. Therefore, 51.4% of LumiraDx™ tests with a negative result were not confirmed by the real time RT-PCR result, which came back as positive, whereas only 1.7% of subjects who tested positive with LumiraDx™ were not confirmed by RT-PCR, which came back as negative (Figure 1).

While the fraction of false negative results among all of the LumiraDx™ tests compared with real time RT-PCR was not significantly associated with gender (*p* = 0.704), a moderately significant difference among the various age groups (0–39 vs. 60–79; *p* = 0.048) was registered. Finally, false negative rates were significantly less common among asymptomatic subjects than both among pauci-symptomatic subjects (*p* = 0.002) and those diagnosed with pneumonia (*p* = 0.009). The false positive fraction did not significatively differ among the various subgroups (Table 1).

It turns out that the LumiraDx™ test, as a whole, presents a sensitivity of 76.3% (70.8–81.8%), high specificity of 94.4% (88.3–100%), PPV of 98.3 (96.4–100%), NPV of 48.6 (39–58.2%), and accuracy of 79.8% (75.1–84.5%), see Table 2.

### 3.2. Impact of Ct Values and Days from Symptoms’ Onset on LumiraDx™ Sensitivity

**Figure 2:**
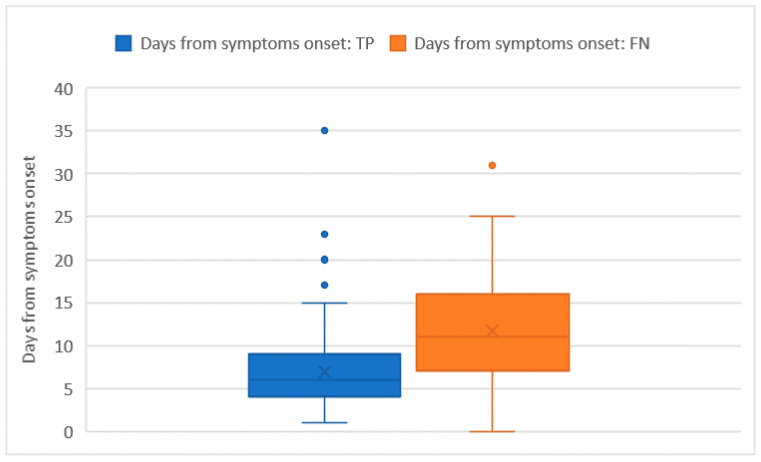
Days from symptoms’ onset distribution among true positive (TP) and false negative (FN) tests.

**Figure 3:**
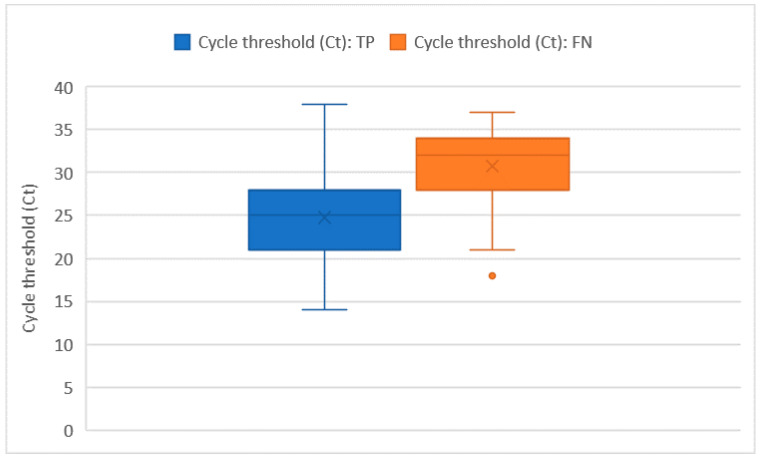
Ct distribution among true positive (TP) and false negative (FN) tests.

Ct values were recorded for all of the 228 RT-PCR assays that tested positive. The average Ct value was 26 ± 5.7 and no significant differences in the Ct distribution were found when comparing different sex or age groups. The Ct values were significantly higher in subjects who were tested as asymptomatic compared with those who had symptoms (32 ± 1.8 vs. 26 + 5.8; *p* = 0.014). However, the comparison may be somewhat affected by the low numbers in the asymptomatic group. Among the 222 tests performed on individuals who developed symptoms, the average number of days that elapsed from symptoms’ onset was 9 ± 6.5 days. The number of days that elapsed from symptoms’ onset showed a moderately positive relation with the Ct value (ρ = 0.23 with *p* < 0.001). In this group of 222 subjects who tested positive with real time RT-PCR and developed symptoms before the testing day, we counted 51 false negative tests with an overall sensitivity of 76.6% (73.7–79.5%), which was not significantly different from the one we computed on the whole tests. As pointed out in Figure 2 and Figure 3, false negative tests were characterised both by the higher number of days (12 ± 6.4 vs. 7 ± 4.4) and higher Ct values (31 ± 4.6 vs. 25 ± 5.3) when compared with true positive (TP) tests (*p* < 0.001 in both cases).

**Figure 4:**
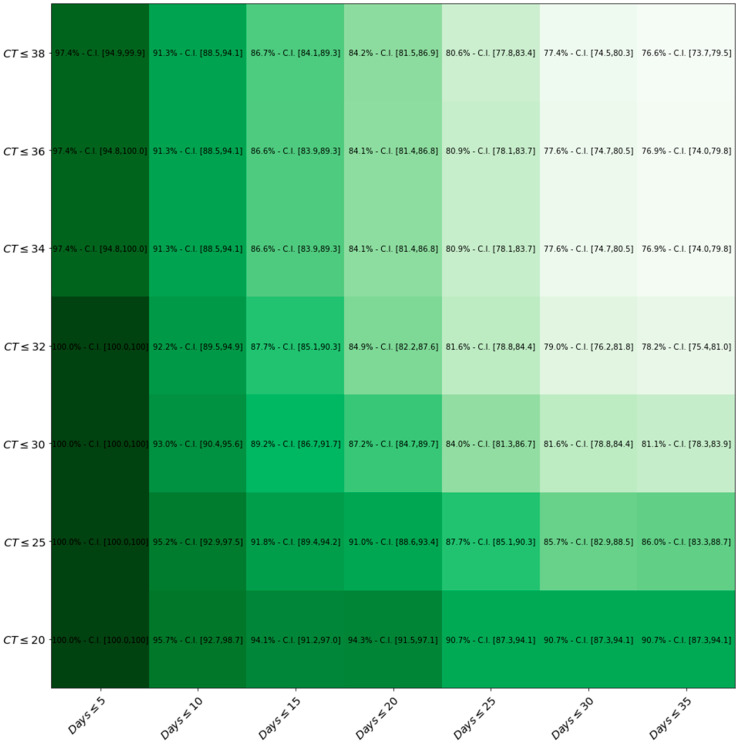
Heat-map displaying sensitivity with respect to both Ct values and Days after symptom onset thresholds. The darker the green, the higher the level of sensitivity of the LumiraDx^TM^ compared to the gold standard RT-PCR.

Figure 4 represents how LumiraDx™ sensitivity changes by the variation of both Ct values and Days.

Table 3 shows the results of multivariate binomial logistic regression, which follows the discordance between the LumiraDx™ outcome and consecutive positive real time RT-PCR assays (i.e., false negative) as a dependent variable and with sex or age group, Ct values, and Days as independent covariates. We did not find a statistically significant correlation between the demographics and LumiraDx™ test results, but a larger delay between the symptoms’ onset and testing date significantly affected the sensitivity of Ag-RTD. Indeed, the risk of a false negative result was greater than four times and then sixteen times when tests were performed between the 11th and 19th day and after the 20th day from symptoms’ onset, respectively. Moreover, a reported viral load (measured by Ct values) between 26 and 31 and >32, was associated with a 4- and 14-fold increased risk of having a negative Ag-RTD test result, respectively.

## 4. Discussion

The aim of this study was to evaluate the performance of the third-generation Ag-RDT LumiraDx™ assay against the real time RT-PCR test results, which was collected in a population tested for SARS-CoV-2 during the second wave of COVID-19 pandemic in Northern Italy. As the SARS-CoV-2 infection is still far from eradication, the introduction in the clinical practice of innovative, rapid, feasible, and accurate new diagnostic tests became an important issue to be addressed. Since the beginning of the pandemic, the number of Ag-RDTs on the market has constantly increased, even though evaluations of the different SARS-CoV-2 Ag-RDTs suggest that their performance may be significantly lower when compared with the gold standard real time RT-PCR test [20]. A study on 100 nasopharyngeal swabs showed that the overall Ag-RDT sensitivity for real time RT-PCR positive samples ranged from 24.3 to 50% [18], whereas in a systematic review and meta-analysis, the pooled Ag-RDT sensitivity was much higher (71.2%) [21].

The introduction of the third-generation Ag-RDT LumiraDx™ assay is likely to change this scenario. In our real-life study, the third generation LumiraDx™ assay demonstrated an overall sensitivity and specificity of 76.3 and 94.4%, respectively.

It has been reported that the first 10-day-period from symptoms’ onset is the most likely window of infectivity for SARS-CoV-2. Interestingly, in our study, the sensitivity was higher than 91% when the analysis was restricted to samples from symptomatic/pauci-symptomatic patients within 10 days from symptoms’ onset. Moreover, the high specificity value was comparable with the pooled specificity of 98% observed throughout all of the Ag-RDT studies, confirming the high PPV of the test.

To date, there is still uncertainty regarding the relationship between SARS-CoV-2 detection, virologic levels, and infectivity during the different stages of the viral trajectory, especially in asymptomatic/pauci-symptomatic patients. It has been estimated that the duration of virus detection in nasopharyngeal secretions of asymptomatic individuals varies largely from 7 to 23 days, even though the presence of viral RNA may not represent the transmissible live virus [22,23]. Indeed, studies examining the infectious period of patients with COVID-19 showed that the virus can be cultured from patients within the first 8 days after symptoms’ onset [24,25,26].

Furthermore, several studies found that viral loads were similar among symptomatic, pauci-symptomatic, and asymptomatic patients [27,28]. The median Ct values did not show any significant differences (24.2 vs. 24.8, respectively) in symptomatic patients with typical symptoms (i.e., fever, cough, and shortness of breath) compared with those displaying atypical symptoms (i.e., chills, malaise, increased confusion, rhinorrhoea/nasal congestion, myalgia, dizziness, headache, nausea, and diarrhoea) [28]. In our study, CT values were significantly higher in asymptomatic subjects compared with symptomatic subjects (32 ± 1.8 vs. 26 + 5.8; *p* = 0.014), even though the comparison may be somewhat affected by the low numbers of asymptomatic subjects in our analysis.

In accordance with our study, there is no clear-cut evidence of a correlation between the false negative outcome and the sex, age or extent of symptoms of the tested patient. By contrast, we showed that the LumiraDx™ sensitivity significantly increases with the rising of both viral loads, which is expressed in terms of lower Ct values, and with the drop in the number of days from symptoms’ onset and testing date. In fact, 100% of sensitivity was detected when symptoms’ onset occurred within 5 days and the Ct threshold was <25. Moreover, it is reasonable to hypothesise that our discordant cases may have underestimated the true duration of infection, failing to recognize the right time of symptoms’ onset.

Indeed, some differences in the sensitivity of LumiraDx™ might also depend on other factors, such as the sample type, specimen quality, handling, and preparation of the test. In our study, the manufacturer’s instructions were strictly observed. In addition, trained health care workers collected the nasal swabs, which were inserted in both nostrils to a distance of about 2 cm, and before the real time RT-PCR nasopharyngeal swab was performed.

Therefore, of note, our study was conducted during the second wave of COVID-19 pandemic, when the UK variant was the predominant viral variant spreading across North-East Italy (>90%) [29].

As a result, it is likely that the LumiraDx™ assay was not impacted by this variant. However, we are not able to assess whether the discordant results can be affected by single nucleotide polymorphisms.

Further novel SARS-CoV-2 virus variants of potential concern with different mutations of the spike protein have recently emerged, such as the Beta (B.1.351), Gamma (P.1), Delta (B.1.617.2), and finally Omicron (B.1.1.529), which are associated with enhanced transmissibility and increased virulence [30]. However, it seems that LumiraDx™ has the ability to detect the virus or whether the diagnostic sensitivity/specificity of this assay is not impaired by viral variants. Recently, the company stated that the LumiraDx™ SARS-CoV-2 Ag test demonstrated similar sensitivity for the detection of the Omicron variant as for other variants during in-house wet testing with the live Omicron virus [31]. However, future clinical and laboratory validation studies should be carried out to explore whether this laboratory testing may perform adequately for the new circulating variants of concern and to inform public health decisions in the following outbreaks.

The study has clear limitations, including the retrospective methodology and limited sample size, which mainly consists of symptomatic subjects. However, we were able to perform all the correlation analysis of demographic factors and symptoms within this group of samples.

Indeed, LumiraDx™ had important advantages, such as rapidity, lower cost, easy-to-use, limited technical skill, and absence of specialised laboratory equipment required, in comparison with molecular testing. In addition, antigenic tests are increasingly used for mass screening purposes in different epidemic phases to control viral transmission, especially in situations where the real time RT-PCR testing capacity is limited. However, solid evidence of the effectiveness of these screenings is still lacking and the low sensitivity found with some antigenic tests may undermine the mass screening efforts. Recently, the ECDC established a minimum performance requirement of at least 80% sensitivity to 90% or greater for subjects with Ct < 25 in symptomatic people (within the first 7 days after symptoms’ onset), and at least 98% specificity for an antigen-based test compared with the real time RT-PCR reference assay [18]. Our study largely meets the sensitivity requirement, whereas the specificity was slightly lower than required. Of note, in our analysis, sensitivity and specificity do not present any significant differences among subjects belonging to different sex or age groups, which are the only two parameters independent of the virus prevalence, whereas sensitivity turns out to be significantly greater (*p* < 0.001) among pauci-symptomatic subjects than among pneumonia-diagnosed individuals, in which it is likely that a longer time of infection has occurred. In fact, in our study, when the antigenic test was carried out between the 11th and 19th day and after the 20th day from symptoms’ onset, the risk of having a false negative test increased by four and sixteen times, respectively.

Furthermore, in epidemiological settings with high prevalence of SARS-CoV-2 infection, the low NPV indicates a higher number of false negative results. In this case, a two-step approach with a real time RT-PCR test is mandatory to avoid missing a COVID-19 diagnosis.

## 5. Conclusions

In conclusion, the above-described characteristics make LumiraDx^TM^ tests suitable for large-scale mass testing and SARS-CoV-2 containment strategies, integrating social distancing, masks usage, serial antigen screenings, and contact tracing. Moreover, the tests are useful for frontline screening in the emergency department to guarantee a fast-track path toward COVID-wards. However, in this setting, the risk of misrecognition of patients with active COVID-19 still exists, and the need for confirmatory real time RT-PCR in symptomatic subjects who test as antigen-negative should not be amended. Given the current availability of different types of COVID-19 testing strategies, LumiraDx^TM^ may not have an antagonist, but a complementary role with the real time RT-PCR test in the pandemic response. Additionally, in accordance with the prevalence of infection in each population and risk mitigation strategies, a two-step approach may be considered and based on country capacity and cost-effectiveness. Furthermore, it may be time- and cost-effective for clinical microbiology laboratories, both to avoid diagnostic delays due to overload and to ensure a high-quality activity for the management of all the other daily microbiological activities.

## Figures and Tables

**Table 1:** Relationship between the test analysis results and clinical variables.

Variables	Test *n*	LumiraDx^TM^ + *n* (%)	LumiraDx^TM^ − *n* (%)	RT-PCR + *n* (%)	RT-PCR − *n* (%)	FN *n* (%)	FP *n* (%)
Sex							
F	105	64 (61)	41 (39)	81 (77.1)	24 (22.9)	18 (43.9)	1 (1.6)
M	177	113 (63.8)	64 (36.2)	147 (83.1)	30 (16.9)	36 (56.3)	2 (1.8)
Age class							
0–39	37	13 (35.1)	24 (64.9)	18 (48.6)	19 (51.4)	6 (25)	1 (7.7)
40–59	88	55 (62.5)	33 (37.5)	69 (78.4)	19 (21.6)	15 (45.5)	1 (1.8)
60–79	115	80 (69.6)	35 (30.4)	106 (92.2)	9 (7.8)	27 (77.1)	1 (1.3)
80+	42	29 (69)	13 (31)	35 (83.3)	7 (16.7)	6 (46.2)	0 (0)
Diagnosis							
Asymptomatic	32	4 (12.5)	28 (87.5)	6 (18.8)	26 (81.3)	3 (10.7)	1 (25)
Pauci-symptomatic	146	117 (80.1)	29 (19.9)	138 (94.5)	8 (5.5)	23 (79.3)	2 (1.7)
Pneumonia	102	55 (53.9)	47 (46.1%)	83 (81.4)	19 (18.6)	28 (59.6)	0 (0)
Neurological symptoms	2	1 (50)	1 (50)	1 (50)	1 (50)	0 (0)	0 (0)
Overall	282	177 (62.8)	105 (3.2)	228 (80.9)	54 (19.1)	54 (51.4)	3 (1.7)

Legend to Table 1. (+): Positive; (−): Negative; F: Female; M: Male; FN: False negative; FP: False positive. FN: LumiraDx™ negative test in the presence of a positive real time RT-PCR test. FP: LumiraDx™ positive test in the presence of a negative real time RT-PCR test.

**Table 2:** Sensitivity, specificity, positive predictive value, negative predictive value, and accuracy of LumiraDx™ test, in accordance with patients’ characteristics.

Attributes of Tested Subject	Sensitivity	Specificity	PPV	NPV	Accuracy	K
Sex						
F	77.8 (68.7–86.9)	95.8 (87.8–100)	98.4 (95.4–100)	56.1 (40.9–71.3)	81.9 (74.5–89.3)	0.59 (0.43–0.74)
M	75.5 (68.5–82.5)	93.3 (84.4–100)	98.2 (95.8–100)	43.8 (31.6–56)	78.5 (72.5–84.5)	0.47 (0.35–0.60)
Age class						
0–39	66.7 (44.9–88.5)	94.7 (84.7–100)	92.3 (77.8–100)	75 (57.7–92.3)	81.1 (68.5–93.7)	0.62 (0.37–0.86)
40–59	78.3 (68.6–88)	94.7 (84.7–100)	98.2 (94.7–100)	54.4 (37.4–71.4)	81.8 (73.7–89.9)	0.58 (0.40–0.76)
60–79	74.5 (66.2–82.8)	88.9 (68.4–100)	98.8 (96.4–100)	22.9 (9–36.8)	75.7 (67.9–83.5)	0.27 (0.11–0.44)
80+	82.9 (70.4–95.4)	100 (100–100)	100 (100–100)	53.8 (26.7–80.9)	85.7 (75.1–96.3)	0.62 (0.36–0.88)
Diagnosis						
Asymptomatic	50 (10–90)	96.2 (88.8–100)	75 (32.6–100)	89.3 (77.8–100)	87.5 (76–99)	0.53 (0.13–0.93)
Pauci-symptomatic	83.3 (77.1–89.5)	75 (45–100)	98.3 (96–100)	20.7 (6–35.4)	82.9 (76.8–89)	0.26 (0.07–0.45)
Pneumonia	66.3 (56.1–76.5)	100 (100–100)	100 (100–100)	40.4 (26.4–54.4)	72.5 (63.8–81.2)	0.42 (0.27–0.57)
Neurological symptoms	100 (100–100)	100 (100–100)	100 (100–100)	100 (100–100)	100 (100–100)	1 (1–1)
Overall	76.3 (70.8–81.8)	94.4 (88.3–100)	98.3 (96.4–100)	48.6 (39–58.2)	79.8 (75.1–84.5)	0.52 (0.42–0.62)

Legend to Table 2. F: Female; M: Male; PPV: Positive predictive value; NPV: Negative predictive value; CI: Confidence interval.

**Table 3:** Summary of multivariate binomial logistic regression (Nagelkerke’s R^2^ = 0.388).

Considered Outcome: False Negative Result of LumiraDx^TM^ Swab					
Model		*p*-Value	adjOR	95% CI adjOR	
				Lower	Upper
Age group	0–39	Ref	-	-	-
	40–59	0.837	0.846	0.173	4.146
	60–79	0.704	1.348	0.289	6.279
	80+	0.424	0.475	0.076	2.952
Sex	Male	0.434	0.716	0.311	1.652
Days	≤10	Ref	-	-	-
	11–19	0.001	4.326	1.816	10.304
	≥20	0.001	16.014	3.116	82.314
Ct	≤25	Ref	-	-	-
	26–31	0.007	3.936	1.463	10.588
	≥32	<0.001	14.482	5.443	38.532

## Data Availability

All of the data used to produce this work are herein reported.
